# Novel isolates expand the physiological diversity of *Prochlorococcus* and illuminate its macroevolution

**DOI:** 10.1128/mbio.03497-23

**Published:** 2024-10-18

**Authors:** Jamie W. Becker, Shaul Pollak, Jessie W. Berta-Thompson, Kevin W. Becker, Rogier Braakman, Keven D. Dooley, Thomas Hackl, Allison Coe, Aldo Arellano, Kristen N. LeGault, Paul M. Berube, Steven J. Biller, Andrés Cubillos-Ruiz, Benjamin A. S. Van Mooy, Sallie W. Chisholm

**Affiliations:** 1 Department of Civil and Environmental Engineering, Massachusetts Institute of Technology, Cambridge, Massachusetts, USA; 2 Department of Marine Chemistry and Geochemistry, Woods Hole Oceanographic Institution, Woods Hole, Massachusetts, USA; 3 Department of Earth, Atmospheric and Planetary Sciences, Massachusetts Institute of Technology, Cambridge, Massachusetts, USA; 4 Department of Biology, Massachusetts Institute of Technology, Cambridge, Massachusetts, USA; University of California, Irvine, Irvine, California, USA; Pontificia Universidad Católica de Chile, Santiago, Chile

**Keywords:** picocyanobacteria, evolutionary biology, marine microbiology, ecophysiology, photosynthetic bacteria

## Abstract

*Prochlorococcus* is a diverse picocyanobacterial genus and the most abundant phototroph on Earth. Its photosynthetic diversity divides it into high-light (HL)- or low-light (LL)-adapted groups representing broad phylogenetic grades—each composed of several monophyletic clades. Here, we physiologically characterize four new *Prochlorococcus* strains isolated from below the deep chlorophyll maximum in the North Pacific Ocean. We combine these physiological properties with genomic analyses to explore the evolution of photosynthetic antennae and discuss potential macroevolutionary implications. The isolates belong to deeply branching low-light-adapted clades that have no other cultivated representatives and display some unusual characteristics. For example, despite its otherwise low-light-adapted physiological characteristics, strain MIT1223 has low chl *b_2_* content similar to high-light-adapted strains. Isolate genomes revealed that each strain contains a unique arsenal of pigment biosynthesis and binding alleles that have been horizontally acquired, contributing to the observed physiological diversity. Comparative genomic analysis of all picocyanobacteria reveals that Pcb, the major pigment carrying protein in *Prochlorococcus*, greatly increased in copy number and diversity per genome along a branch that coincides with the loss of facultative particle attachment. Collectively, these observations support a recently developed macroevolutionary model, in which niche-constructing radiations allowed ancestral lineages of picocyanobacteria to transition from a particle-attached to planktonic lifestyle and broadly colonize the euphotic zone.

**IMPORTANCE:**

The marine cyanobacterium, *Prochlorococcus*, is among the Earth’s most abundant organisms, and much of its genetic and physiological diversity remains uncharacterized. Although field studies help reveal the scope of diversity, cultured isolates allow us to link genomic potential to physiological processes, illuminate eco-evolutionary feedbacks, and test theories arising from comparative genomics of wild cells. Here, we report the isolation and characterization of novel low-light (LL)-adapted *Prochlorococcus* strains that fill in multiple evolutionary gaps. These new strains are the first cultivated representatives of the LLVII and LLVIII paraphyletic grades of *Prochlorococcus*, which are broadly distributed in the lower regions of the ocean euphotic zone. Each of these grades is a unique, highly diverse section of the *Prochlorococcus* tree that separates distinct ecological groups: the LLVII grade branches between monophyletic clades that have facultatively particle-associated and constitutively planktonic lifestyles, whereas the LLVIII grade lies along the branch that leads to all high-light (HL)-adapted clades. Characterizing strains and genomes from these grades yields insights into the large-scale evolution of *Prochlorococcus*. The new LLVII and LLVIII strains are adapted to growth at very low irradiance levels and possess unique light-harvesting gene signatures and pigmentation. The LLVII strains represent the most basal *Prochlorococcus* group with a major expansion in photosynthetic antenna genes. Furthermore, a strain from the LLVIII grade challenges the paradigm that all LL-adapted *Prochlorococcus* exhibit high ratios of chl *b:a_2_*. These findings provide insights into the photophysiological evolution of *Prochlorococcus* and redefine what it means to be a low- vs high-light-adapted *Prochlorococcus* cell.

## INTRODUCTION

The picocyanobacterium *Prochlorococcus* is numerically the most abundant photoautotrophic organism in the global ocean (1). It represents an enormous and genetically diverse collective of cells (2) tuned to thrive under particular environmental conditions (3, 4). *Prochlorococcus* cells have traditionally been divided into high-light (HL)- and low-light (LL)-adapted groups based on their optimal light intensity for growth, which generally corresponds to the depths at which they display their maximal abundance (5–7). Finer-scale niche partitioning among lineages has been linked to other abiotic factors, including temperature, trace metal and inorganic nitrogen acquisition, and vertical mixing (8–11). Over the years since its discovery (12), environmental sequencing efforts throughout the global ocean have continued to uncover novel genetic diversity within the *Prochlorococcus* collective (13–15).

Within the broadly defined HL- and LL-adapted groups of *Prochlorococcus*, monophyletic clades have been designated based on sequence similarity of the 16S/23S rRNA internal transcribed spacer (ITS) sequence (16, 17). Laboratory isolates of *Prochlorococcus* have existed since shortly after its discovery (18, 19), and although more than 100 isolates are now available, environmental sequencing continues to reveal groups within the *Prochlorococcus* collective for which no representative isolates exist. These include the HLIII, HLIV, and HLV lineages typically found in high-nutrient, low-chlorophyll environments (20–23), the HLVI lineage (22), and the basal LLV/AMZ1, LLVI/AMZII, and AMZIII lineages associated with oxygen minimum zones (24, 25). Furthermore, quantitative PCR-based abundance measurements typically underestimate the total number of *Prochlorococcus* cells *in situ* when compared to flow cytometry counts, particularly for samples taken at or near the base of the euphotic zone. Because PCR primers are highly specific and are based on the known diversity of *Prochlorococcus*, this suggests a significant fraction of LL-adapted lineages are not represented in culture collections (9, 26, 27). For some of these uncultivated groups, little is known beyond the fact that they exist, precluding a deeper understanding of the evolutionary history of *Prochlorococcus*.

**Fig 1:**
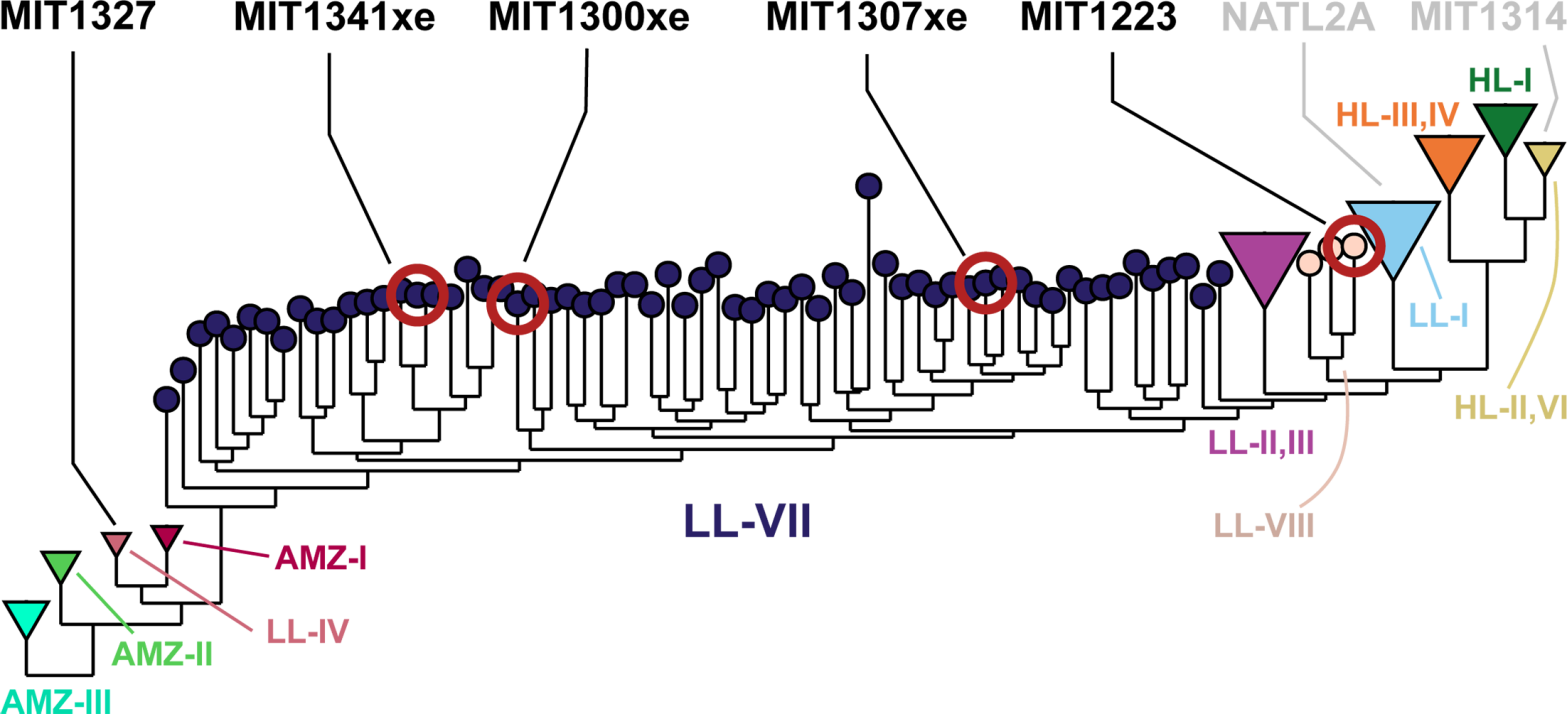
Placement of the isolates used in this study in a phylogeny of 1,200 non-identical *Prochlorococcus* genomes. The genome database contains isolate genomes, MAGs, and SAGs with a wide range of genome completeness, isolation sources, and sequencing methods (see Materials and Methods). The tree was built using FastTree2 on the concatenated alignment of 44 synteny-conserved orthologous genes (Materials and Methods). Major clades are represented by tip color. Novel isolates (black text, red circles) and representative strains (gray text and circles) are indicated on the tree.

One particularly abundant, deeply branching uncultivated group originally called NC1 (27) and later renamed to LLVII (17) is situated phylogenetically between two broad groups—LLIV and LLII/III—associated with different lifestyles (Fig. 1). The transition between these two groups had major implications for the evolution and ecology of ancient *Prochlorococcus*. That is, the more basal LLIV lineage is capable of particle attachment, whereas the LLII/III clade marks the earliest diverging lineage of fully planktonic cells that characterize the rest of the *Prochlorococcus* tree (28). The LLVII group was first reported in a 2009 clone library study where they comprised as much as 61.7% (average 23.1%) of the *Prochlorococcus* sequences detected in the lower euphotic zone (140–160 m) in the North Atlantic and North Pacific oceans (27). This uncultivated group has also been detected at and below 100 m in the Red Sea, where they were the majority (58%) of LL-adapted sequences recovered (29), and in the lower euphotic zone of the western Pacific Ocean and South China Sea (22, 30). Therefore, the physiology and genomic content of the LLVII lineage are particularly intriguing as they could shed light on niche differentiation among LL-adapted groups, as well as inform the macroevolution of *Prochlorococcus* as a whole.

Here, we study the physiology and genomics of deep-branching *Prochlorococcus* lineages and use their properties to refine our synoptic macroevolutionary perspective. We describe a culturing pipeline explicitly targeting the acquisition of deep-branching low-light-adapted lineages, resulting in the isolation of four novel strains belonging to basal lineages. The isolates were physiologically characterized in terms of broad niche-determining traits such as light and temperature optima for growth, photosynthetic capacity, and pigment content. We sequenced their genomes and examined genetic diversity related to pigment biosynthesis and photosynthetic antenna genes of the *pcb* family, which are the primary chlorophyll-binding proteins in *Prochlorococcus*. We conclude by merging the physiological properties and genomic information of these strains with the current macroevolutionary model of forces that drove *Prochlorococcus* diversification in ancient oceans.

## RESULTS AND DISCUSSION

### Phylogenetic placement of isolates

Four *Prochlorococcus* strains were isolated from below the deep chlorophyll maximum and 1% light level (Table 1) using cultivation methods designed to enrich LL-adapted cells. The four strains proved difficult to identify using standard sequence alignment of their ITS regions; they had <70% nucleotide identity to strains from established clades and each other (Fig. S1). These poor alignments precluded the generation of ITS-based phylogenetic trees with reliable bootstrap values, thus we moved to sequence alignments generated using 44 marker genes with conserved synteny and compared them to 1,200 non-identical *Prochlorococcus* isolate genomes, single amplified genomes (SAGs), and metagenome-assembled genomes (MAGs) (Table S2). The four isolates belong to two distinct paraphyletic regions lacking any cultivated representatives (Fig. 1). One of these regions, encompassing strains MIT1300xe, MIT1307xe, and MIT1341xe (where an xe suffix designates xenic cultures, and all other cultures are axenic), falls between the LLII/III and LLIV clades and includes genomes with ITS sequences that resemble those of the NC1 group (later renamed LLVII) discovered in environmental clone libraries (17, 27). Our isolates, along with SAGs from this group (14), confirm hypotheses that these lineages are not monophyletic (22, 27). The fourth isolate (MIT1223) resides in a paraphyletic region located between the LLI and LLII/III clades.

**TABLE 1:** Origin of the *Prochlorococcus* strains described in this work^*a*^

Strain	Clade/ grade	Sample date	Sample depth (m)	Sample temp (^o^C)	Isolation medium	Pre- filtration	Purification method	Light (µmol photons m^−2^ s^−1^)	Notes
MIT1314	HLII	June 2013	150	21.8	Pro2 + 1 µM sodium thiosulfate	Gravity (1.0 µm)	Dilution to extinction	0.3–40	
MIT1223	LLVIII^*a*^	Sept. 2012	175	20.2	16 µM NO_3_^−^ 1 µM PO_4_^3−^ 1/10^th^ Pro99 metals	None	Dilution to extinction	3–19	Additional filtration (0.8 µm)
MIT1300xe	LLVII^*a*^	June 2013	150	21.8	15 µM NO_2_^−^ 1 µM PO_4_^3−^ Pro2/Pro99 trace metals	Gravity (1.0 µm)	Non-axenic	0.3–12	
MIT1307xe	LLVII^*a*^	June 2013	150	21.8	15 µM NO_2_^−^ 1 µM PO_4_^3−^ Pro2/Pro99 trace metals	Gravity (1.0 µm)	Non-axenic	0.3–12	Additional filtration (0.8 µm)
MIT1341xe	LLVII^*a*^	June 2013	150	21.8	15 µM NO_2_^−^ 1 µM PO_4_^3−^ Pro2/Pro99 trace metals	Gravity (1.0 µm)	Non-axenic	0.3–12	Additional filtration (0.8 µm)
MIT1327	LLIV	June 2013	150	21.8	Pro2 + 1 µM sodium thiosulfate	Gravity (1.0 µm)	Dilution to extinction	3–12	

^*a*^ Denotes strains representing lineages with no prior isolates. The isolation location for all strains was station ALOHA in the subtropical North Pacific Ocean (22.75^o^N 158^o^W). The light column indicates the range of irradiance values experienced by the cells during the journey from enrichment to unialgal isolate, and the medium column the nutrient concentrations if they differ from Pro2 and Pro99 media recipes as described in Moore et al. (31).

The fact that all four of these isolates were obtained from the base of the euphotic zone and maintained under low irradiances in the laboratory, along with their phylogenetic placement with previously identified LL-adapted clades, supports hypotheses that these lineages are LL adapted (27, 29). Here, we refer to the paraphyletic region between the LLII/III and LLIV clades (encompassing strains MIT1300xe, MIT1307xe, and MIT1341xe) as the LLVII grade, and the paraphyletic region between the LLI and LLII/III clades (encompassing strain MIT1223) as the LLVIII grade (Fig. 1).

To further understand these novel groups, we analyzed pigment content and growth rate effects of light and temperature on one isolate from each grade, alongside representative HL- and LL-adapted strains from previously described monophyletic clades. Strains MIT1314 (HLII) and MIT1327 (LLIV) were chosen to represent two extremes of the *Prochlorococcus* collective life-history spectrum. MIT1327 (LLIV) represents a deep-branching low-light-adapted strategy that can be particle associated and thrives in the nutrient-rich conditions of the lower water column (28), whereas MIT1314 (HLII) represents a recently diverging high-light-adapted strategy with a streamlined genome that is common in the oligotrophic open ocean (32). To understand how our novel isolates, which are phylogenetically placed between these two representative strains, lie between the two life-history extremes, we focused our experiments on MIT1223 from the LLVIII grade and MIT1300xe as a representative of the LLVII grade.

### Photophysiology

#### Light-dependent growth rates

**Fig 2:**
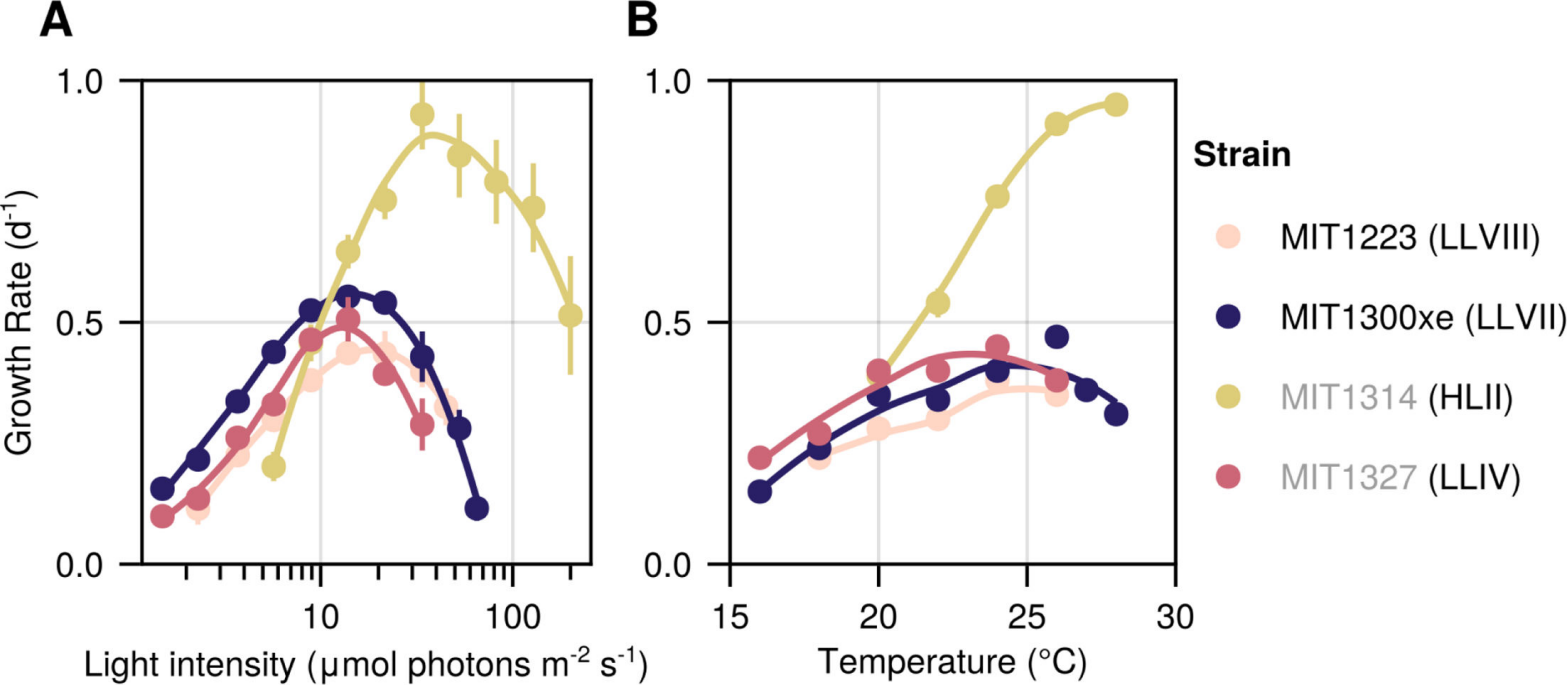
The light- and temperature-dependent growth rate of novel *Prochlorococcus* isolates. Growth rate as a function of light intensity (**A**) and temperature (**B**). Regressions were produced by locally weighted smoothing (LOESS). Circles represent the mean (±standard deviation [SD]) of biological replicate cultures acclimated to each condition (see Tables 2 and 4). Experiments in panel A were conducted at 24^o^C ± 1^o^C, and experiments in panel B were carried out at 76 ± 1 µmol photons m^−2^ s^−1^ for MIT1314; 20 ± 1 µmol photons m^−2^ s^−1^ for MIT1223, MIT1300xe, and MIT1327 on a 14:10 h light:dark cycle. Error bars are smaller than the size of the symbols where not visible. Novel isolate names are marked with black text, whereas previously described strains are listed in gray. The clade/grade designation of each strain is indicated in parenthesis.

Growth rates as a function of light intensity were similar for MIT1223, MIT1300xe, and MIT1327 (LLIV clade) but distinct from MIT1314 (HLII clade)—supporting the notion that the paraphyletic LLVII and LLVIII grades comprise LL-adapted lineages (Fig. 2A). MIT1223, MIT1300xe, and MIT1327 grew at nearly maximal rates at irradiance levels that were too low to support the growth of MIT1314. Conversely, MIT1314 grew well at the highest irradiance (200 µmol photons m^−2^ s^−1^), whereas the other strains did not grow reliably at intensities ≥73 µmol photons m^−2^ s^−1^ (Table 2).

**TABLE 2:** Mean growth rates ± SD for *Prochlorococcus* strains acclimated to different light intensities^*a*^

Strain	Growth rate (day^−1^) at indicated light intensity (µmol photons m^−2^ s^−1^)															
	1.5	2.3	3.7	5.7	8.9	13.9	21.6	33.8	40	45	52.7	65	73	82.2	128	200
MIT1314	ND	ND	ND	0.20 ± 0.03 (12)	0.46 ± 0.04 (20)	0.65 ± 0.04 (20)	0.75 ± 0.04 (18)	0.93 ± 0.07 (17)	—	—	0.84 ± 0.09 (20)	—	—	0.79 ± 0.09 (15)	0.74 ± 0.09 (15)	0.51 ± 0.12 (32)
MIT1223	ND	0.11 ± 0.03 (7)	0.23 ± 0.01 (20)	0.30 ± 0.02 (28)	0.38 ± 0.02 (23)	0.44 ± 0.02 (22)	0.44 ± 0.05 (32)	0.40 ± 0.03 (22)	—	0.32 ± 0.04 (9)	ND	ND	—	ND	—	—
MIT1300xe	0.16 ± 0.00 (10)	0.22 ± 0.00 (10)	0.34 ± 0.01 (12)	0.44 ± 0.01 (14)	0.52 ± 0.01 (16)	0.55 ± 0.02 (18)	0.54 ± 0.02 (16)	0.43 ± 0.05 (18)	—	—	0.28 ± 0.04 (14)	0.12 ± 0.03 (9)	ND	ND	—	—
MIT1327	0.10 ± 0.01 (4)	0.14 ± 0.01 (30)	0.26 ± 0.01 (16)	0.33 ± 0.01 (14)	0.46 ± 0.01 (18)	0.51 ± 0.05 (38)	0.39 ± 0.02 (12)	0.29 ± 0.05 (26)	ND	ND	ND	—	—	—	—	—

^*a*^ ND (not determined) indicates that the strain was tested at this condition. However, a consistent, reproducible growth rate could not be achieved. A dash indicates that growth at this condition was not tested. Parentheses indicate the number of biological replicates included in calculations.

MIT1300xe (LLVII grade) sustained growth at extremely low light intensities, with a growth rate 1.6 times faster than MIT1327 (LLIV clade)—the only other strain capable of growth at 1.5 µmol photons m^−2^ s^−1^—reflecting MIT1300xe’s ability to thrive at the base of the euphotic zone where light limits primary production. MIT1300xe also grew well over a broad range of light intensities (1.5 to 65 µmol photons m^−2^ s^−1^), consistently faster than the other LL-adapted strains (Fig. 2A). We note, however, that MIT1300xe was the only xenic strain tested, and we cannot rule out heterotrophic cells influencing these results due to *Prochlorococcus* mixotrophy (33) and reduction of oxidative stress (34–37). Both MIT1300xe and MIT1223 grew better at higher light intensities than MIT1327, suggesting that members of the LLVII and LLVIII grades may tolerate higher irradiances than those of the LLIV clade. Interestingly, despite originating from depths that typically experience <5 µmol photons m^−2^ s^−1^, all four strains achieved their maximum growth rates at similar light intensities (13.9–33.8 µmol photons m^−2^ s^−1^). However, the maximal growth rate of MIT1314 (HLII clade) was 1.7 times faster than the maximal growth rate of any LL-adapted strain (Table 2). These results reinforce the well-established notion that adaptation to high-light conditions probably occurred only once and relatively late during *Prochlorococcus* evolution (3).

#### Photosynthetic properties

**Fig 3:**
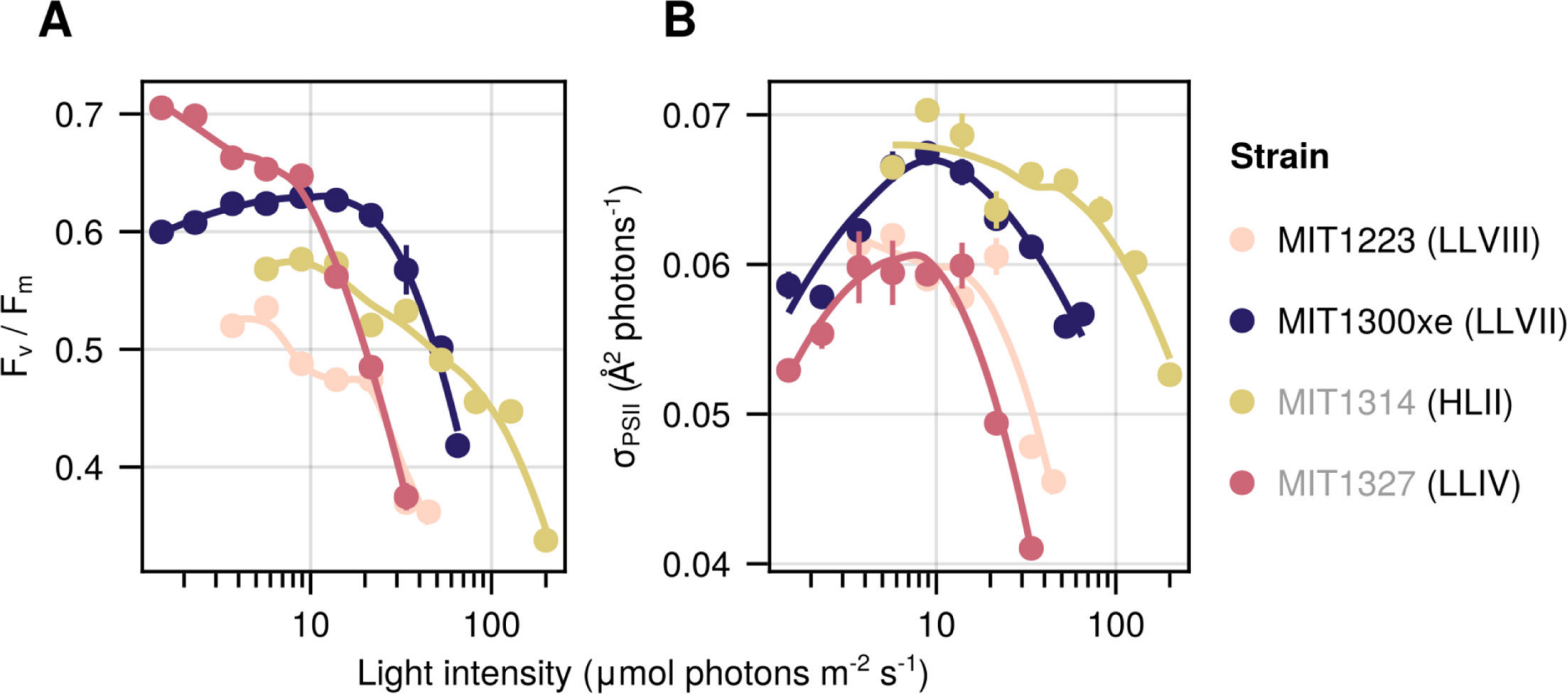
Photosynthetic parameters of novel isolates as a function of growth light intensity. Photosynthetic quantum efficiency (**A**) and functional absorption cross-section (σPSII) (**B**). Regressions produced by locally weighted smoothing (LOESS). Circles represent the mean (±SD) of biological replicate cultures acclimated to each condition sampled over time (*n* = 6 to 10). Error bars are smaller than the size of the symbols where not visible. Novel isolate names are marked with black text, whereas previously described strains are listed in gray. The clade/grade designation of each strain is indicated in parenthesis.

Fast repetition rate fluorometry was used to compare photosynthetic efficiencies and the functional size of light-harvesting antennae among strains when grown at various irradiances. Photosynthetic quantum efficiency (*F*_v_/*F*_m_) generally decreased with increasing light intensity in all four strains, with MIT1327 (LLIV clade) exhibiting the steepest decline, especially at light intensities >8.9 µmol photons m^−2^ s^−1^ (Fig. 3A). MIT1300xe (LLVII grade) had the same photosynthetic quantum efficiency when grown at 1.5 to 21.6 µmol photons m^−2^ s^−1^, but declined at higher intensities, which is in agreement with its consistently high growth rate over this irradiance range when compared to other LL-adapted strains. Interestingly, MIT1300xe is the only strain tested that displayed maximum photosynthetic efficiency when growing at its maximum rate, likely due to the presence of heterotrophs reducing oxidative stress. MIT1327 (LLIV clade) was the most photosynthetic efficient strain at low light intensities, whereas MIT1300xe (LLVII grade) was the most photosynthetic efficient strain at the intermediate light intensities. Photosystem II (PSII) functional absorption cross-sections (σPSII) were largest at intermediate light intensities, with a general decline in σPSII values for all strains at light intensities greater than those at which the maximum growth rate was observed, potentially indicating light-induced damage to PSII at higher irradiances (Fig. 3B). MIT1300xe had larger σPSII values than the other LL-adapted strains at all light intensities, an advantage that is consistent with its faster growth rates.

The photosynthetic properties of MIT1300xe (LLVII) place it as an intermediate in the *Prochlorococcus* collective life-history spectrum, consistent with its phylogenetic placement between the LLIV clade and other ecotypes (Fig. 1). MIT1300xe exhibited the greatest photosynthetic quantum efficiency at intermediate light levels (Fig. 3A) and σPSII values between those of the LLIV and HLII strains at all light intensities (Fig. 3B). This suggests that the photosynthetic apparatus of MIT1300xe may represent a transitional state between LLIV and later-diverging lineages, or that it occupies a niche where these intermediate photosynthetic parameters are advantageous. MIT1223ax (LLVIII), however, exhibited low photosynthetic quantum efficiency despite having σPSII values similar to those of the LLIV strain, suggesting physiological tradeoffs that limit photosynthetic growth efficiency may be a requirement for *Prochlorococcus* cells with a relatively small genome size and low GC content (Table 3) (4) to grow at low irradiances (Fig. 2A).

**TABLE 3:** Genome characteristics and assembly statistics for *Prochlorococcus* strains in this study

Strain	Clade or grade	Assembly size (bp)	% GC	# scaffolds	Average coverage	# coding sequences	JGI genome ID	NCBI BioSample #
MIT1314	HLII	1,704,447	31.2	1	73	1,926	2681813573	SAMN38315734
MIT1223^*a*^	LLVIII	1,795,922	35.7	1	236	1,939	2681813568	SAMN38315731
MIT1300xe^*a*^	LLVII	1,855,146	41.4	1	186	1,965	2681813570	SAMN38315732
MIT1307xe^*a*^	LLVII	2,032,419	39.9	1	317	2,140	2681813572	SAMN38315733
MIT1341xe^*a*^	LLVII	1,937,096	40.1	1	335	2,037	2681813574	SAMN38315735
MIT1327	LLIV	2,587,389	50.3	29^*b*^	204	2,642	2681812949	SAMN04490364

^*a*^ Denotes novel isolates obtained in this study.

^*b*^ N50 for this assembly was 328.4 kbp.

#### Pigment-driven photoacclimation

**Fig 4:**
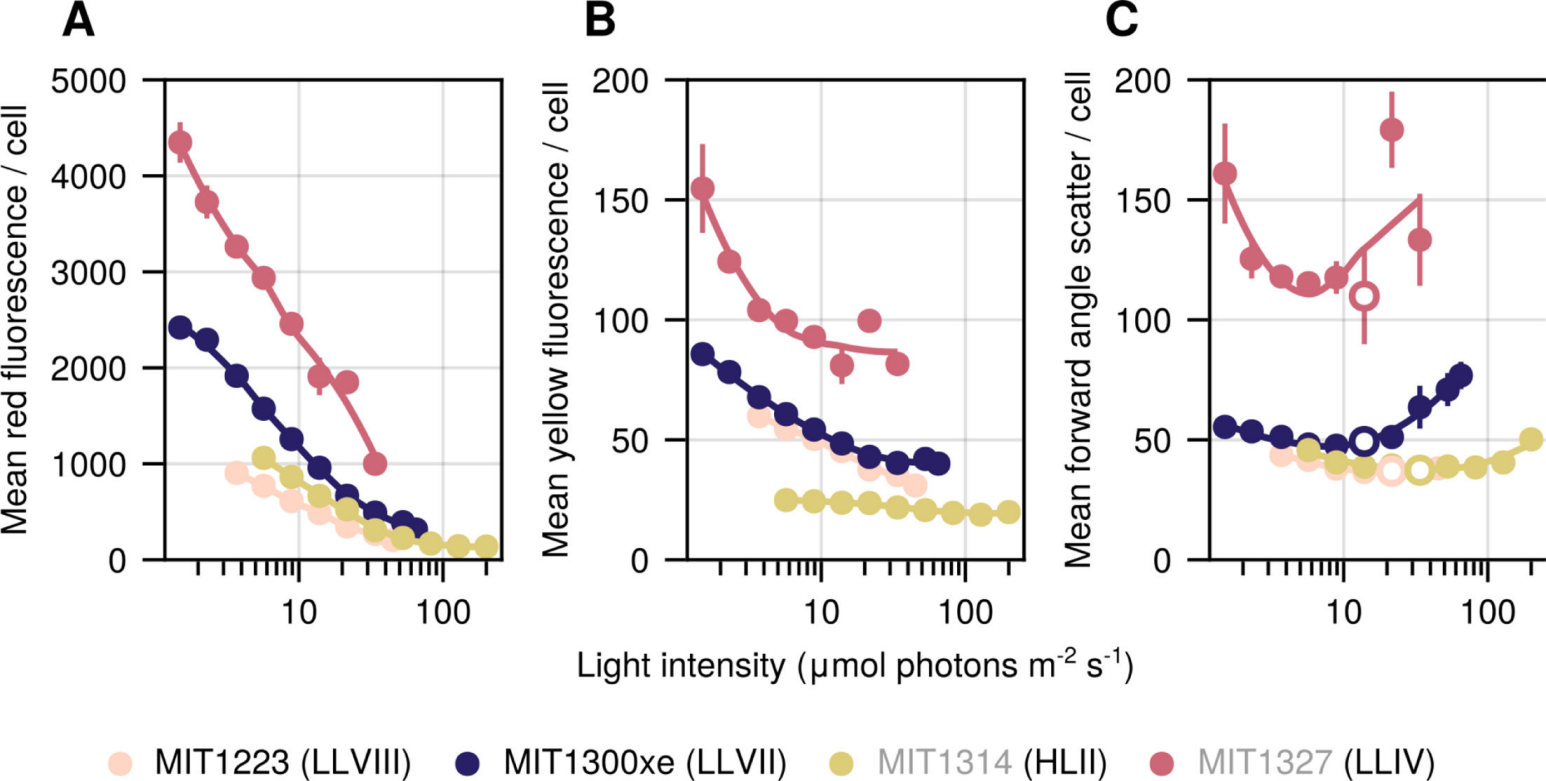
Fluorescence and light scattering properties of novel isolates as a function of growth light intensity. Flow cytometric analyses (**A**) red (chlorophyll) and (**B**) yellow (phycoerythrin) fluorescence per cell, and (**C**) forward light scatter (proxy for cell size). Symbols represent the mean (±SD) of biological replicate cultures acclimated to each condition. Error bars are smaller than the size of the symbols where not visible. Open circles in panel **C** denote the light level at which maximum growth rate was achieved for each strain. Novel isolate names are marked with black text, whereas representative strains are listed in gray. The lineage of each strain is indicated in parenthesis.

*Prochlorococcus* cells tune their pigment content to irradiance (5, 19), often revealed as an anticorrelation between chlorophyll (red) fluorescence per cell and growth irradiance in flow cytometric signatures. HL- and LL-adapted strains are known to display different slopes, where pigment fluorescence decreases more drastically for LL-adapted cells with increasing light intensity (5, 32). We found this slope to be intermediate for MIT1300xe (LLVII grade), which is similar to previous observations of cells from the LLII/III clade (Fig. 4A; see also reference 32). Surprisingly, despite being adapted to grow at low light intensities, MIT1223 (LLVIII grade) appeared similar to MIT1314 (HLII clade) in this regard (Fig. 4A). In terms of yellow fluorescence, which is mainly mediated by phycoerythrin in *Prochlorococcus*, the HLII strain (MIT1314), which lacks phycoerythrin biosynthetic potential (38), represents the lowest extreme, whereas the LLIV strain (MIT1327) represents the highest. The novel LLVII and LLVIII isolates are intermediate in terms of slope and total yellow fluorescence/cell levels, recapitulating their evolutionary location between the basal LLIV and the more recently emerged HL-adapted clades (Fig. 4B).

All four strains tended to increase in size with increasing irradiance (Fig. 4C). As expected, based on their clade and genome size, MIT1327 (LLIV clade) cells were the largest cells at all light intensities (39). The forward angle scatter per cell of MIT1223 (LLVIII grade) was similar to that of the HLII strain at all irradiance levels (Fig. 4C), providing further evidence that MIT1223 does not follow all canonical descriptions of a LL-adapted *Prochlorococcus* cell. These results provide potential clues about the period of evolutionary tinkering that occurred during the divergence of LLIV and other *Prochlorococcus*—suggesting that changes to the physiology and pigments that occurred as cells adapted to a planktonic lifestyle may have occurred in distinct stages.

To explore whether or not the unexpected properties of MIT1223’s photophysiology—i.e., chlorophyll (red) fluorescence and forward angle scatter per cell—were unique to the LLVIII grade, we examined *Prochlorococcus* strain NATL2A from the LLI clade (6, 40, 41) acclimated to four irradiance levels that fall within the middle of the range tested for the other strains. NATL2A grew faster than the other LL-adapted strains (MIT1223 and MIT1327) at higher light intensities (≥26.3) but slower than the HL-adapted strain (MIT1314) (Fig. S2A). This finding supports the notion that LLI clade *Prochlorococcus* can tolerate higher irradiance levels than other LL-adapted strains—perhaps due in part to the presence of a photolyase gene and an abundance of high light-inducible genes (13, 42). Despite its unique light-dependent growth rates (Fig. S2A), the *in vivo* absorption spectra, median chlorophyll (red) fluorescence per cell, and median forward angle scatter per cell of NATL2A (LLI clade) were quite similar to those of MIT1223 (LLVIII grade) and MIT1314 (HLII clade), and distinct from MIT1327 (LLIV clade) (Fig. S2B, C and E). The median yellow (primarily phycoerythrin) fluorescence per cell signature of NATL2A was lower than MIT1223 and similar to that of MIT1314 (Fig. S2D). Although a detailed pigment analysis of *Prochlorococcus* strains from the LLI clade is needed to confirm these absorption and flow cytometry results, our experiments indicate that despite its phylogenetic placement among LL-adapted clades, NATL2A, like MIT1223, possesses many physiological characteristics that are traditionally associated with HL-adapted strains (e.g., lower red and yellow fluorescence per cell, reduced forward angle light scatter, and likely lower chl *b:a_2_* ratios as indicated by *in vivo* absorption spectra).

**Fig 5:**
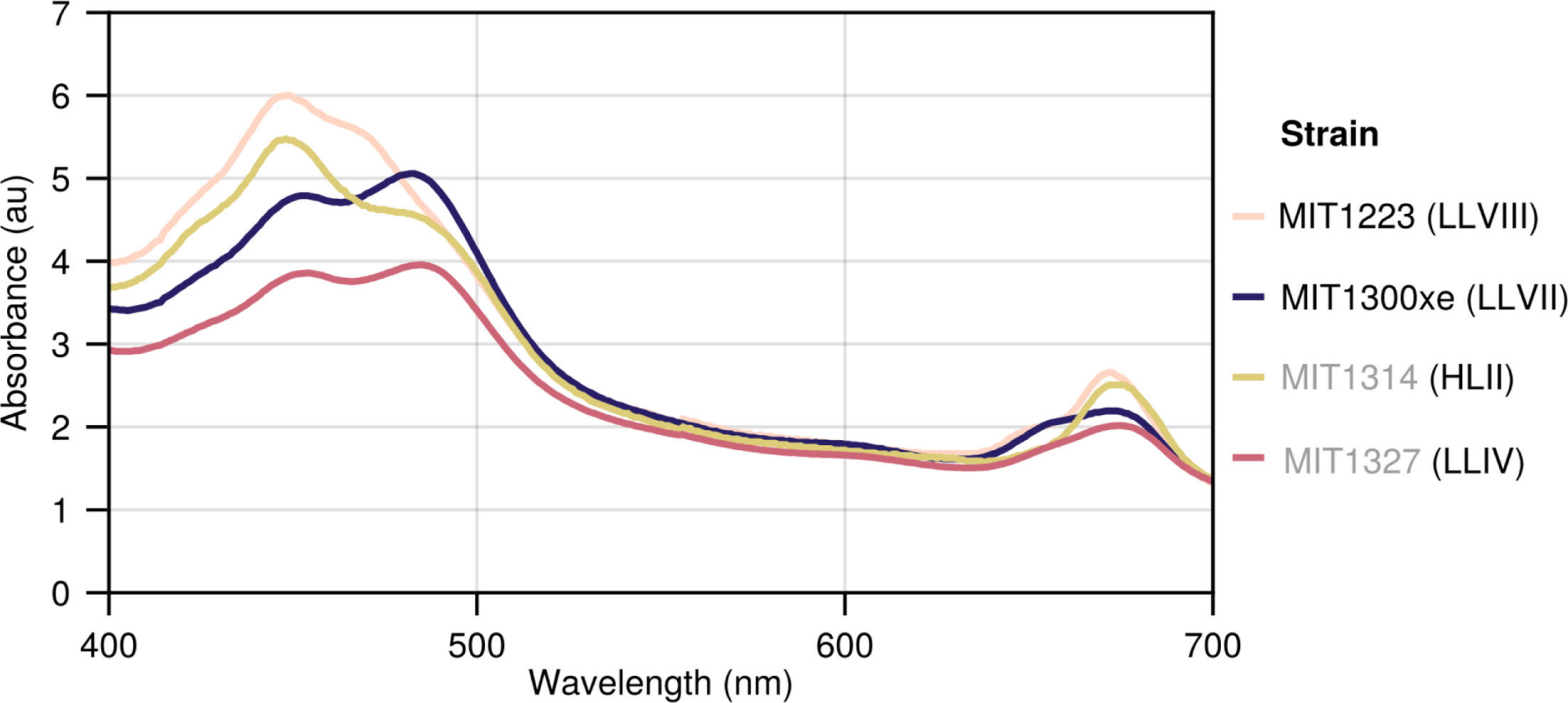
*In vivo* absorption spectra of novel isolates compared to representatives from established LL- and HL-adapted clades. Spectra measured for log phase cultures growing at 5.7 µmol photons m^−2^ s^−1^. Novel isolate names are marked with black text, whereas representative strains are listed in gray. The lineage of each strain is indicated in parenthesis.

*In vivo* absorption spectra provide further evidence that the pigmentation of MIT1223 is unique among the LL-adapted strains. *Prochlorococcus* cells have traditionally been divided into two major groups based on chlorophyll content with HL-adapted cells exhibiting low total chl *b* (chl *b_1_*+ chl *b_2_*) to chl *a_2_* ratios and LL-adapted cells exhibiting high chl *b:a_2_* ratios (6, 19, 32). Comparing spectra for all strains grown at the same low light intensity, the second peak at ca. 480 nm (chlorophyll *b_2_*) is visible for MIT1327 (LLIV) and MIT1300xe (LLVII), with a higher absorbance than the peak at ca. 450 nm (chlorophyll *a_2_*) (Fig. 5). In contrast, the chl *b_2_* peak for MIT1314 (HLII) is lower than its chl *a_2_* peak, matching the canonical definition of HL-adapted strains (6, 19). The absorption spectra of MIT1223 (LLVIII) are anomalous for a LL-adapted strain, with no discernible chl *b_2_* peak and a faint peak at ca. 470 nm—likely due to a combination of zeaxanthin and α/β-carotene (5)—providing further support for atypical pigmentation in the LL-adapted MIT1223 strain.

**Fig 6:**
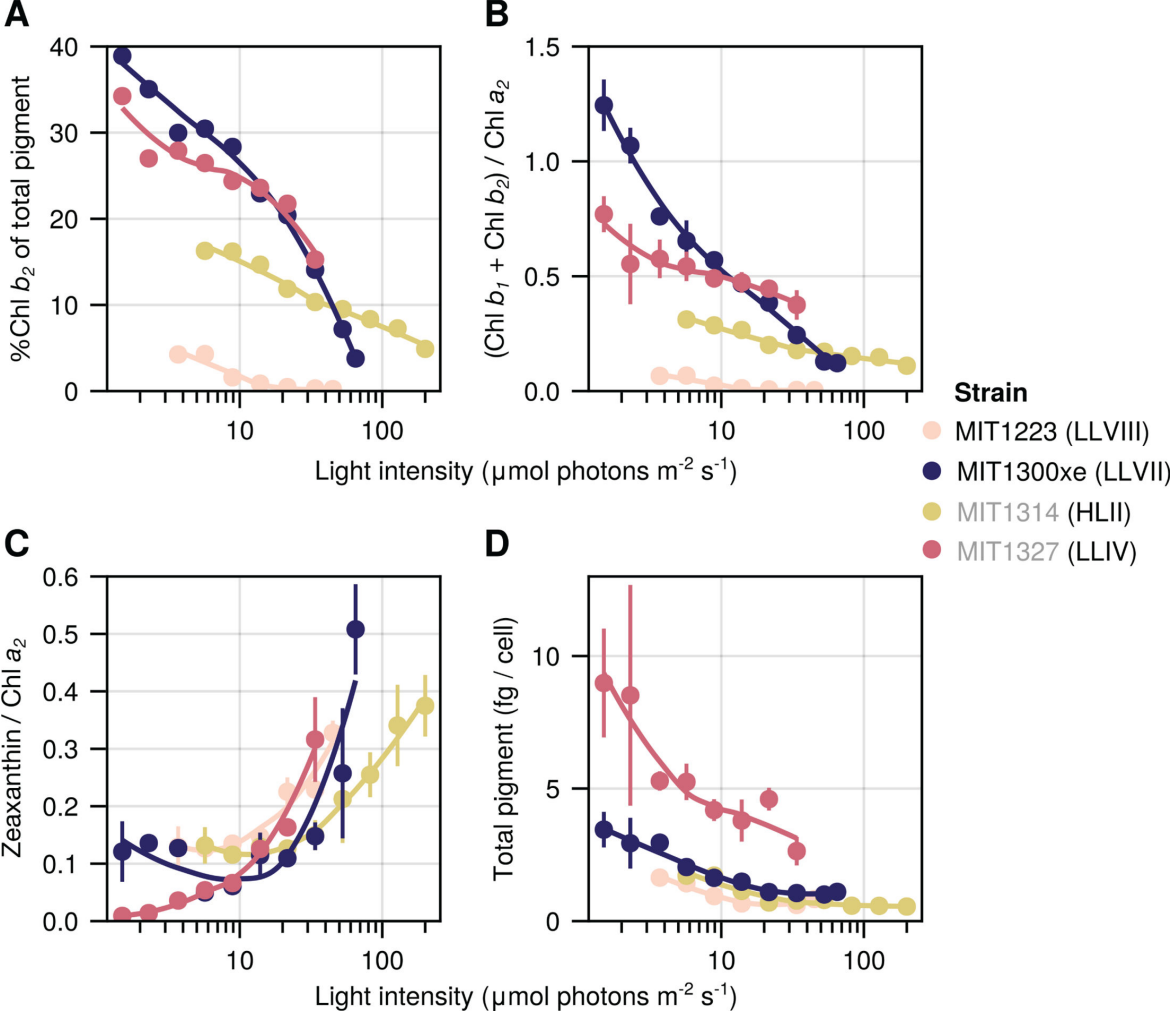
Chlorophyll content of the novel isolates as a function of growth light intensity. Percent of chlorophyll *b_2_* from the total extractable pigment (**A**), chlorophyll *B*/*a_2_* ratios (**B**), zeaxanthin/*a_2_* ratios (**C**), and total extractable pigment per cell (**D**). Values are means (± SD) of biological replicate cultures acclimated to each condition sampled over time (*n* = 2 to 7). Lines represent local LOESS fits. Novel isolate names are marked with black text, whereas representative strains are listed in gray. The lineage of each strain is indicated in parenthesis.

We next analyzed pigments using high-performance liquid chromatography (HPLC) and mass spectrometry (MS), which confirmed the *in vivo* absorption spectra and provided additional information (Fig. S3 and S4). Strikingly, the chl *b_2_* values for MIT1223 were the lowest of the four strains, including HL-adapted MIT1314 (Fig. 6A). LL-adapted strains MIT1327 (LLIV) and MIT1300xe (LLVII) contained appreciable amounts of chl *b_2_* relative to the total measurable pigment content (4%–39%), especially at lower light intensities, whereas the relative abundance of chl *b_2_* in MIT1314 (HLII) was lower at all light intensities where both strains could grow (5%–16%; Fig. 6A).

As expected, the ratio of chl *b:a_2_* decreased with increasing light intensity for all strains. MIT1300xe (LLVII) showed ratios >1 when grown at low intensities (Fig. 6B), similar to previous findings for strains from the LLII and LLIV clades (19, 32, 43). These high ratios likely facilitate growth at very low irradiances (32). However, the strains tested here still do not have ratios as high as those reported for wild *Prochlorococcus* cells (44), thus the influence of complex heterotrophic partnerships on this ratio may be at play here; the highest chl *b:a_2_* ratios *in vitro* have been measured for xenic cultures (5, 31; this study). MIT1300xe also demonstrated a remarkable capacity for photoacclimation with a range of chl *b:a_2_* ratios spanning more than one order of magnitude (Fig. 6B). MIT1327 (LLIV) had the next highest chl *b:a_2_* ratios; however, these ratios were <1, even when growing under the lowest intensities (Fig. 6B). As expected from previous studies, MIT1314 (HLII) had lower chl *b:a_2_* ratios than MIT1300xe and MIT1327 at all light levels with less photoacclimation over the range of light levels tested (Fig. 6B). The chl *b:a_2_* ratios of MIT1223 (LLVIII) were the lowest of any strain tested—as much as 80-fold lower than MIT1300xe at the same light level. MIT1223 displayed little photoacclimation, and chl *b:a_2_* ratios for this strain dropped below 0.01 at the highest light intensities (Fig. 6B). MIT1223 is, therefore, an exception to the long-standing paradigm that LL-adapted strains contain higher ratios of chl *b:a_2_* than HL-adapted strains.

Ratios of zeaxanthin to chl *a_2_* generally increased with increasing light intensity, supporting its photoprotective role (5, 19) (Fig. 6C). MIT1327 had the highest total extractable pigment per cell (2.6–9.0 fg/cell) compared to the other three strains tested, and MIT1223 had values at or below those for MIT1314 when grown at the same irradiance (Fig. 6D). Other primary pigments detected in all four strains consisted of α/β-carotene (indistinguishable with our method), pheophytin *a_2_*, Mg 3,8 divinyl pheoporphyrin (DVP) *a_5_*, and an unknown carotenoid (Fig. S3 to S5), all of which are consistent with previous reports analyzing the pigment content of *Prochlorococcus* cells (19, 45). Based on its exact mass, the unknown carotenoid has a sum formula of C_40_H_56_O, which matches with both cryptoxanthin and β-carotene 5,6-epoxide. However, the MS^2^ fragment data do not appear to match published spectra for either compound (Fig. S5). It has been previously suggested that *Prochlorococcus* may contain cryptoxanthin (45), but due to the MS^2^ results we obtained, we do not feel comfortable making a definitive identification, and additional information such as nuclear magnetic resonance analysis is needed to elucidate the structure of this carotenoid. We also putatively identified the compound all-*trans*-retinal in all four strains due to an exact match (retention time and mass) to a standard (Fig. S6). However, this identification remains ambiguous, given that the abundance was low, and therefore MS^2^ spectra could not be obtained. We feel the putative identification of this compound is worth noting; however, as the potential ability of *Prochlorococcus* to produce all-*trans*-retinal could have important implications for syntrophic interactions in the marine environment, given the importance of this molecule for proteorhodopsin-based phototrophy (46, 47).

### Thermophysiology

Temperature-dependent growth rates also revealed similar trends for MIT1223, MIT1300xe, and MIT1327—which were quite distinct from that of MIT1314—suggesting the paraphyletic LLVII and LLVIII regions comprise lineages of *Prochlorococcus* that are adapted to colder conditions found deeper in the water column near the base of the euphotic zone—the waters from which they were isolated (Fig. 2B; Table 1). Although all of the LL-adapted strains grew much slower than the HL-adapted strain at temperatures ≥24°C, MIT1300xe was still able to grow at the highest temperature at which the HL-adapted strain was able to grow, 2°C warmer than the upper limit of the other LL-adapted strains (Table 4). Again, we cannot rule out the potential influence of heterotrophic bacterioplankton, but note that MIT1327 (LLIV clade) grew faster than MIT1300xe at temperatures ≤24°C, despite being axenic. Consistent with the light physiology experiments, MIT1223 generally grew slower than the other strains regardless of temperature, but it could tolerate colder conditions than the HL-adapted strain (Table 4).

**TABLE 4:** Mean growth rates ± SD for *Prochlorococcus* strains acclimated to different temperatures^*a*^

Strain	Growth rate (day^−1^) at indicated temperature (^o^C)									
	15	16	18	20	22	24	26	27	28	29
MIT1314	—	—	ND	0.39 ± 0.00 (2)	0.54 ± 0.03 (3)	0.76 ± 0.00 (2)	0.91 ± 0.01 (2)	—	0.95 ± 0.00 (2)	ND
MIT1223	—	ND	0.22 ± 0.01 (2)	0.28 ± 0.01 (2)	0.30 ± 0.00 (3)	0.38 ± 0.01 (3)	0.35 ± 0.01 (2)	ND	—	—
MIT1300xe	ND	0.15 ± 0.00 (2)	0.24 ± 0.01 (2)	0.35 ± 0.01 (2)	0.34 ± 0.00 (3)	0.40 ± 0.00 (2)	0.47 ± 0.01 (2)	0.36 ± 0.00 (2)	0.31 ± 0.00 (2)	ND
MIT1327	ND	0.22 ± 0.00 (2)	0.27 ± 0.00 (2)	0.40 ± 0.00 (2)	0.40 ± 0.00 (3)	0.45 ± 0.01 (2)	0.38 ± 0.00 (2)	ND	—	—

^*a*^ ND (not determined) indicates that the strain was tested at this condition. However, a consistent, reproducible growth rate could not be achieved. A dash indicates that growth at this condition was not tested. Parentheses indicate the number of biological replicates included in calculations.

### Photosynthetic antennae evolution

Genomes of the four isolates ranged from 1.8 to 2.0 Mb in size with 35.7%–41.4% GC, intermediate to HL-adapted strains and those from the LLIV clade (4) (Table 3). We examined light-harvesting genes to place these new genomes in context and explore a genetic basis for their observed physiological properties. Unlike other cyanobacteria, most *Prochlorococcus* ecotypes do not produce phycobilisomes as light-harvesting antennae, and instead rely on pigment-binding *pcb* proteins to channel photons to their photosystem II core (48). This property gives *Prochlorococcus* its distinct fluorescence and absorption profiles and is one of the hallmark adaptations that allowed this group to colonize the entire photic zone (49). Because these antenna proteins are the major pigment-containing elements in their photosystems, a plausible hypothesis is that the photophysiological features of these novel LLVII/VIII isolates are due—at least in part—to unique combinations of pigment and antenna. Thus, we focused our genomic comparisons on these features.

**Fig 7:**
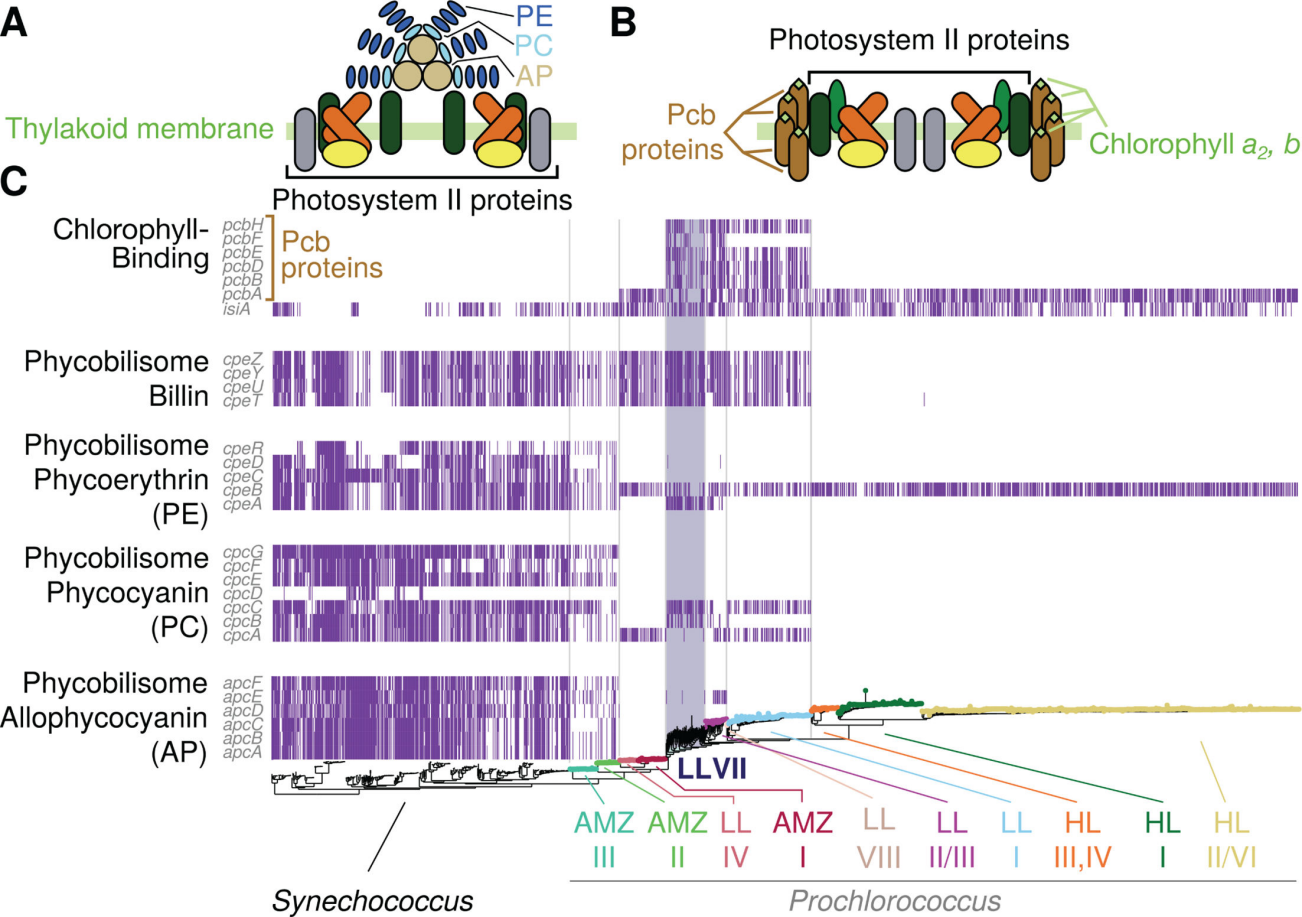
Genomic analysis of photosynthetic pigment biosynthesis and antenna genes across picocyanobacteria. Simplified structure of the phycobilisome (**A**) and prochlorosome (**B**) with the major pigments indicated by their abbreviations. (**C**) Distribution of light-harvesting antenna genes across the phylogeny of marine picocyanobacteria. The tree at the bottom represents the picocyanobacterial phylogeny as in Figure 1, with *Synechococcus* lineages added (see Materials and Methods). Major lineages are depicted by colored text and corresponding tip point colors, except for *Synechococcus* which has no colored tips, and LLVII which is highlighted with a colored background in the tree and in the matrix. Each row in the matrix represents a single KEGG ortholog (and *isiA*), and colored bars signify the presence of a gene (row) in a genome (column). Vertical lines separate Syn, AMZ-III/II, LLIV/AMZ-I, LLVII, LLI-III, and HL groups. The variation in fine-scale presence/absence patterns observed within clades is most likely due to the inclusion of partial genomes in our database.

In agreement with previous reports (25), our analysis shows that most picocyanobacteria encode the iron-inducible *isiA* gene, and *Synecococcus* and the basal *Prochlorococcus* groups (AMZII and AMZIII) encode the full suite of phycobilisome genes but no *pcb* genes. *pcbA,* the first *pcb* gene to diverge from *isiA*, first appears in the ancestor of the LLIV/AMZ-I radiation (Fig. 7C). A comparison of the genomes from 1,691 *Synechococcus* and *Prochlorococcus* picocyanobacteria (Methods) reveals a major expansion in *pcb* gene copy number and diversity for the LLVII grade that is conserved in later-branching LL-adapted clades but lost in the ancestor of all HL-adapted cells, which only contain *pcbA* (Fig. 7). The different ecotypes of *Prochlorococcus* have distinct repertoires of antenna genes, suggesting the observed genetic changes occurred in the ancestor of the group, and that selection has been strong enough to retain these changes.

Examining the evolution of individual antenna genes reveals a complicated history of divergence and horizontal transfer of multiple genes (Fig. S7 and S8). In some cases (such as *pcbD*), MIT1223 (LLVIII) shares alleles with LLVII strains, whereas in others (e.g., *pcbB*), it is closer to LLI strains. Interestingly, MIT1223 never seems to share antenna-related alleles with HL strains (Fig. S7 and S8), suggesting that its “HL-like” characteristics are due to other genetic determinants such as regulation of pigment biosynthesis.

These findings give further support for the important role photophysiology played in the evolution of *Prochlorococcus* (17), in that major radiations involved significant changes to the configuration of the photosynthetic machinery—more specifically in the light-harvesting apparatus. This began with the complete loss of phycobilisomes in the ancestor of LLIV/AMZI, replaced by the more streamlined *pcb* apparatus (Fig. 7, diagram and total number of proteins needed by each antenna type). This transition was capitalized upon by later-diverging lineages, showing remarkable variation in the diversity and copy number of *pcb* and pigment biosynthesis genes.

### Macroevolutionary implications

Unique photophysiology and a dramatic expansion of antenna proteins in a distinct region of the *Prochlorococcus* phylogeny could have broader macroevolutionary implications. Ancestral state reconstructions of *Prochlorococcus* metabolism have suggested its last common ancestor thrived throughout the euphotic zone, with metabolic innovations later producing descendants better able to grow at lower nutrient concentrations near the surface (50). It was proposed that this led to a process of evolutionary niche partitioning in which the emergence of new lineages drew down nutrient levels near the surface, displacing surviving diversity from ancestral lineages to deeper water (50), leading to modern depth-dependent ecotype abundance distributions (9–11, 42). Although other work has focused on different constraints influencing the macroevolution of *Prochlorococcus* (e.g., references 25, 51–54), the “depth-displacement” model outlined above helps to clarify the distribution of the nitrate reductase gene, *narB,* in picocyanobacteria. The *narB* gene is found in most marine *Synechococcus* and was recently confirmed in the low-light-adapted AMZ clades of *Prochlorococcus* (25) after previous detection in oxygen-deficient subsurface waters of the Eastern Pacific Ocean (55). The *narB* gene appears to have been further retained in more recently emerged *Prochlorococcus* lineages such as the LLI, HLI, and HLII clades, whereas *narB* is absent in other low-light-adapted clades (56). Analyses of the genomic location of *narB* further indicate that this gene was vertically inherited from the last common ancestor of *Prochlorococcus* to the LLI and HL clades (56). Taken together, this is consistent with a view in which ancestral *Prochlorococcus* populations had a broad distribution in the water column and encoded the proteins to use nitrate, an ability then lost in some lineages as they were displaced to deeper waters (50) where it was no longer advantageous to assimilate nitrate (56).

The depth-displacement model was recently expanded following the finding that *Synechococcus*, as well as basal lineages of *Prochlorococcus*, attach to and use chitin, a highly abundant form of particulate organic carbon derived from arthropod exoskeletons (28, 57). This extended model suggests that ancestral marine picocyanobacteria colonized the ocean while attached to chitin particles, as this helped them survive the low-nutrient, high-UV conditions of the open ocean (28). Over millions of years, cells then acquired adaptations that allowed them to make the transition to a fully planktonic lifestyle, restricting chitin utilization to the deep euphotic zone where light is limiting. The loss of the chitin utilization trait and the postulated transition to a constitutive planktonic lifestyle occur along the branch that separates LLIV and all other *Prochlorococcus* clades (28)—the same branch that has the LLVII grade as its foundation (Fig. 1). The dramatic expansion of *pcb* genes in the LLVII grade could thus reflect the tipping point toward a fully planktonic lifestyle, as the loss of access to particle-derived organic carbon inputs could well exert a strong selective pressure on light harvesting. Indeed, Pcb proteins enhance the light-gathering capacity of photosystems (58) and, in *Prochlorococcus* strains that carry multiple *pcb* copies, total Pcb levels are higher compared to strains with fewer copies (59). The distinct physiology and unexpected pigmentation of the LLVIII isolate provide an additional opportunity to begin further investigation of the eventual emergence of the HL clades that dominate the upper euphotic zone today.

### Conclusions

The four isolates described here help resolve two diverse, largely unexplored regions within the LL-adapted ecotypes of the *Prochlorococcus* collective. The isolates belong to two distinct paraphyletic regions in an area of the *Prochlorococcus* phylogeny with long branch lengths (i.e., the paraphyletic LLVII and LLVIII regions, along with the LLII/III clade). The area is flanked by three monophyletic clades (LLI, AMZI, and LLIV) containing lineages with shorter branch lengths. The inclusion of single-cell genomes and metagenomes from field samples provides culture-independent evidence supporting these placements.

A representative from the LLVII grade displayed photophysiology properties and genomic characteristics that were generally intermediate to representatives from the LLIV and HLII clades—two extremes of the *Prochlorococcus* collective life-history spectrum. A representative from the LLVIII grade displayed many photophysiology properties indicative of a LL-adapted strain. Its pigmentation, genome size, and GC content, however, resemble that of a HL-adapted strain, thus challenging several long-standing paradigms regarding light adaptation among the *Prochlorococcus* collective. Preliminary work with strain NATL2A (LLI) indicates these anomalies may also extend to members of the LLI clade. Our findings blur the distinction between these historically defined ecotypes and suggest pigmentation and pigment-driven photoacclimation are likely unreliable indicators of *Prochlorococcus*’ optimal irradiance levels for growth and ecotype designation.

*Prochlorococcus* lineages from regions of the phylogenetic tree with longer branch lengths—and therefore higher genetic divergence from nearby lineages—may reflect divergent evolution among LL-adapted *Prochlorococcus* in which a confluence of environmental variables, including light, has driven substantial diversification from ancestral strains. This is in contrast to the more rapid adaptive radiation events observed within the monophyletic LLI and LLIV clades, which may be a product of finer-scale niche partitioning among lineages. As the LLVII and LLVIII grades are further examined in the context of evolutionary transitions and the rise of *Prochlorococcus*, the high diversity and long branch lengths contained within these groups may reflect prolonged periods of evolutionary tinkering that occurred during those transitions. Future investigations into the unique photophysiological features we have identified in individual strains, and the genomic and genetic diversity within the grades as a whole, could thus provide important new clues as to how *Prochlorococcus* evolved to dominate the extant oceans.

## MATERIALS AND METHODS

### Isolation and identification of novel *Prochlorococcus* strains

*Prochlorococcus* strains mentioned in this study (Table 1) were isolated from the lower euphotic zone at Station ALOHA in the subtropical North Pacific Ocean (22.75^o^N 158^o^W). Strain MIT1223 was isolated from 175 m on 12 September 2012, during the HOE-DYLAN cruise, whereas all other strains were isolated from 150 m on 2 June 2013, during the HOE-PhoR I cruise. The presence and complexity of *Prochlorococcus* populations were monitored over time by flow cytometry (BD/Cytopeia Influx). The seawater base of all media was sterilized by 0.2-µm filtration followed by autoclaving and was prepared in acid-washed autoclaved borosilicate glass or polycarbonate tubes.

MIT1223 was derived from an initial enrichment of unfiltered raw seawater amended with 16 µM NaNO_3_, 1 µM NaH_2_PO_4_.H_2_O, and the trace metal mix used in Pro99 medium (31) reduced 10-fold. The enrichment was initially maintained for ca. 1 year by serial passage at 22–24°C and ≤8 µmol photons m^−2^ s^−1^ on a 14:10 h light:dark cycle, whereas inorganic nutrient conditions were increased to 80 µM NaNO_3_ and 5 µM NaH_2_PO_4_.H_2_O before acclimating to a modified Pro99 medium (800 µM NH_4_Cl replaced by 800 µM NaNO_3_) and transitioning to higher irradiance—19 ± 2 µmol photons m^−2^ s^−1^ on a 14:10 h light:dark cycle. Several months later, flow cytometry monitoring revealed the presence of larger picoeukaryote-like cell populations in addition to several *Prochlorococcus* populations. The enrichment was subsequently passed through a 0.8-µm polycarbonate filter (Nucleopore, Whatman/GE) by gentle gravity filtration to remove the larger cells. This enrichment was then maintained by serial passage for another 15 months before flow cytometry monitoring revealed an apparent unialgal *Prochlorococcus* population, and sequencing revealed the ITS sequence of what we now refer to as strain MIT1223, a member of the paraphyletic LLVIII grade.

Enrichments for *Prochlorococcus* conducted on the HOE-PhoR I cruise in 2013 were prepared as previously described (60, 61). MIT1314 and MIT1327 were derived from an enrichment amended at sea with Pro2 medium nutrients (31) plus 1 µM thiosulfate, whereas MIT1300xe, MIT1307xe, and MIT1341xe were derived from an enrichment from the same original 1.0 µm filtered water sample amended with nitrite as the sole exogenous nitrogen source (Table 1). These enrichments and their derivatives from serial passage were maintained at 22–25°C on a 14:10 h light:dark cycle for 3 months before transitioning to continuous light. The enrichments were maintained at very low irradiances to select for LL-adapted cells, with maximum light exposure of ca. 1 µmol photons m^−2^ s^−1^.

After an initial incubation period of 1 month, the enrichment that ultimately yielded MIT1314 and MIT1327 was transferred into Pro2 medium plus 1 µM thiosulfate to match the original amendment based on Hawaii surface seawater collected during HOE PhoR I. Two weeks later, a subculture was transitioned to 0.2 µm filtered and autoclaved Sargasso seawater amended with Pro99 nutrients (31). Enrichments were maintained by serial passage in their respective media—MIT1314 derives from the Pro99 line, whereas MIT1327 derives from the Pro2 line. After 4 months of serial passage, dilution to extinction in ProMM medium was applied to the Pro2 enrichment, yielding the LLIV clade strain MIT1327 (60). After 5 months in the laboratory, a subculture of the Pro99 enrichment was moved to higher light (10 ± 2 µmol photons m^−2^ s^−1^) to select for a subset of the *Prochlorococcus* diversity observed by flow cytometry. After 6 mo at this irradiance, ITS screening revealed the HLII clade strain MIT1314.

After an initial incubation period of 7 wk, the enrichment yielding MIT1300xe, MIT1307xe, and MIT1341xe was maintained by serial passage in Pro99 medium (31) with a Sargasso seawater base. After 4 months, replicate subcultures were established and maintained as separate transfer series, with one ultimately resulting in MIT1307xe and the other resulting in MIT1300xe and MIT1341xe. After 1 year in the laboratory, these enrichments still contained diverse *Prochlorococcus* populations based on flow cytometry and ITS PCR sequencing. Once again, a subculture was transitioned to higher light (10 ± 2 µmol photons m^−2^ s^−1^), and after 1 year, ITS screening revealed the sequence of what we now refer to as strain MIT1300xe. Flow cytometry monitoring of the two enrichments maintained below 1 µmol photons m^−2^ s^−1^ revealed the presence of LLIV-like *Prochlorococcus* populations and additional *Prochlorococcus* populations with distinct fluorescence signatures. These enrichments were then passed through a 0.8-µm polycarbonate filter (Nucleopore, Whatman/GE) by gentle gravity filtration in an attempt to remove the larger LLIV-like cells. After several months, flow cytometry monitoring revealed an apparent unialgal *Prochlorococcus* population in each, and ITS screening revealed the sequence of what we now refer to as strains MIT1307xe and MIT1341xe.

MIT1314, MIT223, and MIT1327 were purified using a high-throughput dilution to extinction method, and the purity of these isolates was confirmed by flow cytometry and a suite of purity test broths (ProAC, ProMM, and MPTB) as previously described (40, 60, 61). Dilution to extinction purification was repeatedly attempted on the other strains without success.

Initial strain identification was performed by PCR screening of the ITS gene. In brief, 1 mL of culture (ca. 10^6^ to 10^8^ cells) was centrifuged for 15 to 30 min at 16,000 *× g* to form a pellet. The majority of supernatant was removed before centrifugation again at 16,000 *× g* and removal of all residual seawater before resuspending the pellet in 25 to 100 µL Tris-HCl (pH 8.0). Cells were then lysed by boiling at 95°C for 10 min before centrifugation for 5 min at 16,000 × *g* at 4°C to pellet cell debris. The supernatant was removed and used directly in a PCR screen with primers (ITS-F: 5′-CCGAAGTCGTTACTYYAACCC-3′ and ITS-R: 5′-TCATCGCCTCTGTGTGCC-3′), which target the internal transcribed spacer (ITS) region of *Prochlorococcus* as previously described (16, 62, 63).

### Growth rate experiments

The physiological response of four *Prochlorococcus* isolates (MIT1314, MIT1223, MIT1300xe, and MIT1327) to light availability and temperature was measured in a series of growth experiments. All cultures were maintained in Pro99 medium with a Sargasso surface seawater base in acid-washed autoclaved borosilicate glass tubes.

For light-dependent growth experiments (except NATL2Aax), duplicate tubes of each strain were acclimated to target light levels under continuous light (Sylvania cool white bulbs screened to produce target intensity levels) and were monitored weekly using a light meter (LI-COR LI-250A) connected to a spherical micro quantum sensor (Walz US-SQS/L). Ninety-five percent of irradiance measurements were within 10% of the target light level for strains MIT1223, MIT1300xe, and MIT1327, and within 16% of the target light level for MIT1314 (Table S1; Fig. S9). Temperature was monitored daily and held stable at 24°C ± 1°C. Balanced growth was confirmed by examining growth rates over time at each condition. Attempts were made to acclimate each strain to increasingly higher and lower light intensities until reproducible growth could not be achieved. Growth was monitored daily by flow cytometry (see below) and bulk chlorophyll fluorescence (10AU model, Turner Designs) for 4–6 d. Bulk chlorophyll fluorescence readings were blank subtracted using the mean of triplicate medium blanks, and any readings corresponding to lag phase or early onset of stationary phase were identified by eye and removed prior to calculating mean growth rates (Table 2). For light-dependent growth experiments with NATL2Aax, triplicate tubes were acclimated to four target light levels (10, 20, 45, and 96 ± 0.5 µmol photons m^−2^ s^−1^) on a 14:10 h light:dark cycle. Growth was monitored daily by flow cytometry (see below) and bulk chlorophyll fluorescence (10AU model, Turner Designs) for 8–16 d, and mean growth rates were calculated from blank-subtracted fluorescence readings taken in exponential phase.

For temperature-dependent growth experiments, duplicate tubes of each strain were acclimated to specific temperatures (±0.5°C) and grown (76 ± 1 µmol photons m^−2^ s^−1^ for MIT1314; 20 ± 1 µmol photons m^−2^ s^−1^ for MIT1223, MIT1300xe, and MIT1327) on a 14:10 h light:dark cycle. Acclimation was defined as described above, and attempts were made to acclimate each strain to increasingly higher and lower temperatures until reproducible growth could not be achieved. Growth was monitored daily, and mean growth rates were calculated as described above (Table 4).

### Flow cytometry and fast repetition rate fluorometry

Cell concentrations were determined using a Guava Technologies easyCyte 12HT flow cytometer (EMD Millipore). Samples were diluted in sterilized Sargasso seawater to ensure <500 cells µL^−1^ to avoid coincidence counting and were run with only the blue (488 nm) excitation laser enabled for maximum power. Technical duplicate *Prochlorococcus* populations were resolved for each biological sample based on their red (695/50 nm) emission parameters for enumeration. A fluorescent bead reagent (easyCheck Beads, EMD Millipore) was run daily as a reference and to verify instrument performance. Median forward angle scatter, chlorophyll (695/50 nm), and yellow (583/26 nm) fluorescence per cell were determined for each population.

The maximum quantum efficiency of photochemistry in PSII (Fv/Fm) and the functional absorption cross-section of PSII (σPSII) were measured using fast repetition rate fluorometry (FRRF) on a FIRe fluorometer instrument (Satlantic) as previously described (64). Samples were stored in the dark for 15 min prior to loading into the instrument, and Pro99 medium was used for blank measurements.

### Absorption spectra and pigment measurements

*In vivo* absorption spectra were obtained using a Beckman DU 800 spectrophotometer (Beckman Coulter Inc.) in an absorbance scan mode from 400 to 700 nm at 1.0-nm intervals and a scan speed of 1,200 nm/min, and Pro99 medium was used for blank measurements.

For pigment sampling, duplicate *Prochlorococcus* cultures, as well as technical duplicates from each biological replicate, were filtered using vacuum filtration (ca. −200 mm Hg) onto 47-mm diameter 0.2-µm hydrophilic Durapore filters (Millipore). Samples were immediately flash frozen and stored in liquid nitrogen (−196°C) until processing. Pigments were extracted using a modified Bligh and Dyer protocol (65) with DNP-PE-C_16:0_/_C16:0_-DAG (2,4-dinitrophenyl phosphatidylethanolamine diacylglycerol; Avanti Polar Lipids, Inc., Alabaster, AL, USA) used as an internal standard. Filter blanks and *Prochlorococcus* growth media blanks were extracted and analyzed alongside samples. The total lipid extract was analyzed by reverse-phase HPLC MS on an Agilent 1200 HPLC coupled to a Thermo Fisher Exactive Plus Orbitrap high-resolution mass spectrometer (Thermo Fisher Scientific, Waltham, MA, USA). HPLC and MS conditions were as previously described (66, 67). In brief: 20 µL was injected onto a C8 Xbridge HPLC column (particle size 5 µm, length 150 mm, width 2.1 mm; Waters Corp., Milford, MA, USA). Lipids were eluted at a flow rate of 0.4 mL min^−1^ using the following gradient with eluent A (water with 1% 1M ammonium acetate and 0.1% acetic acid) and eluent B (70% acetonitrile, 30% isopropanol with 1% 1M ammonium acetate and 0.1% acetic acid): 45% A was held for 1 min, from 45% A to 35% A in 4 min, from 25% A to 11% A in 8 min, from 11% A to 1% A in 3 min with an isocratic hold until 30 min. Finally, the column was equilibrated with 45% A for 10 min. Electrospray ionization (ESI) source settings were Spray voltage, 4.5 kV (+), 3.0 kV (–); capillary temperature, 150°C; sheath gas and auxiliary gas, both 21 (arbitrary units); and heated ESI probe temperature, 350°C. Mass data were collected in full scan while alternating between positive and negative ion modes. Pigments were identified using retention time, accurate molecular mass, and isotope pattern matching of proposed sum formulas in full-scan mode and tandem MS (MS^2^) fragment spectra of representative compounds. For each MS full scan, up to three MS^2^ experiments targeted the most abundant ions with N2 as collision gas. The scan range for all modes was 100–1,500 *m/z*. The mass spectrometer was set to a resolving power of 140,000 (full width at half-maximum [FWHM] at *m/z* 200), leading to an observed resolution of 75,100 at *m/z* 875.5505 of our internal standard, DNP-PE. Exact mass calibration was performed by weekly infusing a tune mixture. Additionally, every spectrum was corrected using a lock mass, providing real-time calibrations. To validate the accuracy and reliability quantification, quality control samples of known composition spiked with lipid standards were interspersed with the samples as described previously (67). Pigment abundances were corrected for the relative response of commercially available standards. The abundances of chlorophylls and their associated compounds were corrected for the response of a chlorophyll *a* standard and carotenoid pigments using a β-carotene standard. All standards were purchased from Sigma-Aldrich (St. Louis, MO, USA). Individual response factors were obtained from external standard curves by triplicate injection of a series of standard mixtures ranging from 0.15 to 40 pmol on column per standard. Our method’s use of external standards was validated in a study that compared lipid quantitation against internal, isotope-labeled standards (68). Data were corrected for differences in extraction efficiency using the recovery of the DNP-PE internal standard.

### Phylogenetic analysis

Maximum likelihood trees were reconstructed using FastTree (69) from 44 concatenated single-copy core proteins that have conserved local synteny (Table S2) in 1,691 non-redundant picocyanobacterial genomes (1,201 *Prochlorococcus* and 490 *Synechococcus*). Four thousand five hundred twelve genomes matching either *Prochlorococcus* or *Synechococcus* in their description were downloaded from NCBI and IMG on 7 December 2022. To filter out very closely related sequences, we used FastANI (70) to calculate all pairwise whole-genome average nucleotide identity (ANI) values. From these results, a network was created where nodes represent genomes and links represent >99.99% identity over 95% of the longer genome in the pair. This network had 1,961 connected components, and the longest genome from each component was picked as the representative, resulting in the final genome set used in downstream analysis. Gene calling was performed using Pyrodigal v2.0.2 (71), and protein sequences were clustered using MMseqs2 v14-7e284 (72) based on 50% identity and 80% coverage. Initial protein homology clusters were then split based on their genomic surroundings into groups that share at least 3 of the 10 protein families in their immediate genomic vicinity. The final protein clusters, therefore, share both homology and local synteny. We found 44 final clusters that are present in >50% of analyzed genomes and calculated the protein alignment of each protein cluster using mafft v7.310 (73). We then transformed the protein alignments to nucleotide alignments and concatenated all individual alignments to one long alignment that was used for phylogenetic inference with FastTree v2.1.10 (69).

### Genome sequencing and analysis

Closed genome sequences of MIT1223, MIT1300xe, MIT1307xe, and MIT1341xe were obtained as previously described for MIT1314 (61). In brief, genomic DNA was extracted from concentrated cells using the MasterPure complete DNA and RNA purification kit (Epicentre) and was sequenced using P6 chemistry on a RS II instrument (Pacific Biosystems). Library construction and sequencing were performed at the University of Massachusetts Medical School Deep Sequencing and Molecular Biology Core Laboratory. The average insert range for libraries constructed using a standard SMRTbell Template Prep Kit 1.0 and Sequencing Primer v3 (Pacific Biosciences) was 21–28 kb. Reads were assembled *de novo* into a single contig for each strain using the RS_HGAP_Assembly.2 protocol within the SMRT Analysis software (v2.0; Pacific Biosciences) before circularization using the Geneious sequence analysis package (V7.1, Biomatters) and polishing using Quiver and the RS_Resequencing.1 protocol within the SMRT Portal software. The average coverage for each assembly ranged from 73X to 335X (Table 3). The draft genome of MIT1327 was obtained as previously described (60) and consists of 29 scaffolds with an N50 of 328.4 kbp and an average coverage of 204X (Table 3). All genomes have been deposited in GenBank at the National Center for Biotechnology Information (NCBI) and the Joint Genome Institute’s Integrated Microbial Genomes (IMG) system (see Table 3 for IDs) and annotated using the IMG Annotation Pipeline version 4 (74, 75). Sequenced isolates were included in the genome collection described above.

The proteomes of all genomes were annotated using the eggnog v6 database (76). Input proteins were competitively aligned to the database using MMseqs2 with a minimum identity of 30% and 50% coverage. The annotations of the best annotated hit of each input protein were then transferred to the input protein. For the analysis in Figure 7, antenna proteins in the “Photosynthesis Proteins” kegg pathway (ko00194) were extracted based on their eggnog-derived KEGG Orthology numbers, and their presence/absence in genomes was tabulated and plotted on the phylogenetic tree. For each KO, the phylogenetic tree was built by aligning KO members using mafft and building a tree using FastTree as above. Gene trees were rooted using the minimal ancestor deviation criterion (77).

## Data Availability

All genomes have been deposited in GenBank at the National Center for Biotechnology Information (NCBI) and the Joint Genome Institute’s Integrated Microbial Genomes (IMG) system under the numbers in Table 3. Raw data, including nucleotide and protein alignments, as well as data to recreate Fig. 7, were uploaded to https://zenodo.org/doi/10.5281/zenodo.12604418.

## SUPPLEMENTAL MATERIAL

Fig. S1 mbio.03497-23-s0001.eps MAFFT alignment of ITS regions.

Fig. S2 mbio.03497-23-s0002.eps Photophysiology comparison with NATL2A (LLI clade).

Fig. S3 mbio.03497-23-s0003.eps HPLC-Orbitrap-MS chromatograms of identified pigments in *Prochlorococcus* MIT1314.

Fig. S4 mbio.03497-23-s0004.eps Molecular structures of pigments identified in *Prochlorococcus* strains.

Fig. S5 mbio.03497-23-s0005.eps HPLC-Orbitrap-MS chromatogram of an unknown pigment in *Prochlorococcus* MIT1314.

Fig. S6 mbio.03497-23-s0006.eps HPLC-Orbitrap-MS chromatogram of an all-*trans*-retinal standard in *Prochlorococcus* MIT1314.

Fig. S7 mbio.03497-23-s0007.eps Trees of picocyanobacterial genes that contain the pfam PF00421 (Photosystem II protein) domain.

Fig. S8 mbio.03497-23-s0008.eps Trees of phycobilisome-associated genes.

Fig. S9 mbio.03497-23-s0009.eps Percent deviation from target light level for irradiance measurements taken over the course of light-dependent growth experiments.

Supplemental material mbio.03497-23-s0010.docx Table S1, Table S2, and legends for supplemental figures.

ASM does not own the copyrights to Supplemental Material that may be linked to, or accessed through, an article. The authors have granted ASM a non-exclusive, world-wide license to publish the Supplemental Material files. Please contact the corresponding author directly for reuse.
