# Supplementary material for: Novel isolates expand the physiological diversity of Prochlorococcus and illuminate its macroevolution
Source: mBio. 2024 Oct 18;15(11):e03497-23. doi: 10.1128/mbio.03497-23 (PMC11559063; doi:10.1128/mbio.03497-23)
Supplement: Supplemental material — Table S1, Table S2, and legends for supplemental figures. [file mbio.03497-23-s0010.docx]

| Target light level | Median light level | Mean  light level | Standard deviation | Minimum value | Maximum value | CV | Mean %  of target | 2.5th percentile | 97.5th percentile |
| --- | --- | --- | --- | --- | --- | --- | --- | --- | --- |
| 1.5 | 1.5 | 1.5 | 0.1 | 1.0 | 1.6 | 5.5 | 98.0 | 1.3 | 1.6 |
| 2.3 | 2.3 | 2.3 | 0.1 | 1.8 | 2.8 | 5.3 | 98.8 | 1.9 | 2.5 |
| 3.7 | 3.7 | 3.7 | 0.2 | 2.9 | 4.5 | 5.7 | 99.6 | 3.2 | 4.0 |
| 5.7 | 5.7 | 5.7 | 0.3 | 4.8 | 6.5 | 4.7 | 100.0 | 5.2 | 6.3 |
| 8.9 | 8.8 | 8.7 | 0.4 | 7.3 | 9.8 | 4.2 | 98.2 | 7.7 | 9.3 |
| 13.9 | 13.8 | 13.7 | 0.6 | 11.6 | 16.2 | 4.3 | 98.8 | 12.3 | 14.7 |
| 21.6 | 21.4 | 21.4 | 1.1 | 16.6 | 26.0 | 5.3 | 99.0 | 18.5 | 23.2 |
| 33.8 | 33.7 | 33.5 | 1.6 | 27.0 | 39.1 | 4.7 | 99.2 | 29.4 | 36.4 |
| 40.0 | 39.6 | 39.2 | 0.9 | 38.0 | 40.1 | 2.2 | 97.9 | 38.0 | 40.1 |
| 45.0 | 44.5 | 44.2 | 2.0 | 38.6 | 48.0 | 4.5 | 98.3 | 39.2 | 47.1 |
| 52.7 | 52.0 | 51.9 | 2.6 | 44.0 | 66.5 | 4.9 | 98.4 | 46.9 | 55.7 |
| 65.0 | 63.2 | 63.6 | 3.0 | 57.0 | 76.0 | 4.8 | 97.8 | 57.6 | 69.8 |
| 73.0 | 75.3 | 75.1 | 3.3 | 70.0 | 81.0 | 4.4 | 102.8 | 70.2 | 80.8 |
| 82.2 | 82.0 | 81.5 | 5.7 | 65.7 | 104.4 | 7.0 | 99.2 | 69.2 | 90.3 |
| 128.0 | 129.5 | 128.0 | 8.7 | 108.0 | 151.2 | 6.8 | 100.0 | 109.5 | 149.2 |
| 200.0 | 192.0 | 191.5 | 10.2 | 166.0 | 212.0 | 5.3 | 95.7 | 170.2 | 210.9 |

**Table S1. Summary statistics for light measurements (µmol photons m^-2^ s^-1^) taken over the course of the light-dependent growth rate experiments**. Measurements were taken weekly and minor adjustments were made to maintain target levels. Two of the target light levels listed above (40.0 and 73.0 µmol photons m^-2^ s^-1^) were used for acclimation purposes – no samples were taken at these levels.

| **KEGG Orthology ID** | ***gene / Protein Name*** | **Description** |
| --- | --- | --- |
| **K00033** | *gnd, gntZ /* PGD | 6-phosphogluconate dehydrogenase [EC 1.1.1.44 1.1.1.343] |
| **K00053** | *ilvC* | ketol-acid reductoisomerase [EC 1.1.1.86] |
| **K00133** | *asd* | aspartate-semialdehyde dehydrogenase [EC 1.2.1.11] |
| **K00208** | *fabI* | enoyl-[acyl-carrier protein] reductase I [EC 1.3.1.9/1.3.1.10] |
| **K00284** | *gltS /* GLU | glutamate synthase (ferredoxin) [EC 1.4.7.1] |
| **K00297** | *metF /* MTHFR | methylenetetrahydrofolate reductase (NADH) [EC 1.5.1.54] |
| **K00505** | TYR | tyrosinase [EC 1.14.18.1] |
| **K00548** | *metH /* MTR | 5-methyltetrahydrofolate--homocysteine methyltransferase [EC 2.1.1.13] |
| **K00615** | *tktA, tktB* | transketolase [EC 2.2.1.1] |
| **K00616** | *talB, talA  /* TALDO1 | transaldolase [EC 2.2.1.2] |
| **K00789** | *metK /* MAT | S-adenosylmethionine synthetase [EC 2.5.1.6] |
| **K00966** | *GMPP* | mannose-1-phosphate guanylyltransferase [EC 2.7.7.13] |
| **K00975** | *glgC* | glucose-1-phosphate adenylyltransferase [EC 2.7.7.27] |
| **K01139** | *spoT* | GTP diphosphokinase guanosine-3',5'-bis(diphosphate) 3'-diphosphatase [EC 2.7.6.5 3.1.7.2] |
| **K01262** | *pepP* | Xaa-Pro aminopeptidase [EC 3.4.11.9] |
| **K01414** | *prlC* | oligopeptidase A [EC 3.4.24.70] |
| **K01662** | *dxs* | 1-deoxy-D-xylulose-5-phosphate synthase [EC 2.2.1.7] |
| **K01714** | *dapA* | 4-hydroxy-tetrahydrodipicolinate synthase [EC 4.3.3.7] |
| **K01872** | *alaS /* AARS | alanyl-tRNA synthetase [EC 6.1.1.7] |
| **K01874** | *metG /* MARS | methionyl-tRNA synthetase [EC 6.1.1.10] |
| **K01887** | *argS /* RARS | arginyl-tRNA synthetase [EC 6.1.1.19] |
| **K01956** | *carA /* CPA1 | carbamoyl-phosphate synthase small subunit [EC 6.3.5.5] |
| **K02112** | *atpD /* ATPF1B | F-type H+ Na+-transporting ATPase subunit beta [EC 7.1.2.2 7.2.2.1] |
| **K02358** | *tuf /* TUFM | elongation factor Tu |
| **K02492** | *hemA* | glutamyl-tRNA reductase [EC 1.2.1.70] |
| **K02707** | *psbE* | photosystem II cytochrome b559 subunit alpha |
| **K02899** | *rpmA /* RP-L27, MRPL27 | large subunit ribosomal protein L27 |
| **K02992** | *rpsG /* RP-27, MRPS7 | small subunit ribosomal protein S7 |
| **K03040** | *rpoA* | DNA-directed RNA polymerase subunit alpha [EC 2.7.7.6] |
| **K03043** | *rpoB* | DNA-directed RNA polymerase subunit beta [EC 2.7.7.6] |
| **K03106** | *ffh /* SRP54 | signal recognition particle subunit SRP54 [EC 3.6.5.4] |
| **K03495** | *gidA, mnmG /* MTO1 | tRNA uridine 5-carboxymethylaminomethyl modification enzyme |
| **K03587** | *ftsI* | cell division protein FtsI (penicillin-binding protein 3) [EC 3.4.16.4] |
| **K04567** | *lysS /* KARS | lysyl-tRNA synthetase, class II [EC 6.1.1.6] |
| **K05581** | *ndhJ* | NAD(P)H-quinone oxidoreductase subunit J [EC 7.1.1.2] |
| **K05896** | *scpA* | segregation and condensation protein A |
| **K06118** | *sqdB /* SQD1 | UDP-sulfoquinovose synthase [EC 3.13.1.1] |
| **K06158** | ABCF3 | ATP-binding cassette, subfamily F, member 3 |
| **K07769** | *nblS, dfr, dspA* | hik33 two-component system, OmpR family, sensor histidine kinase NblS [EC 2.7.13.3] |
| **K08482** | *kaiC* | circadian clock protein KaiC |
| **K09458** | *fabF /* OXSM | CEM1 3-oxoacyl-[acyl-carrier-protein] synthase II [EC 2.3.1.179] |
| **K11532** | *glpX /* SEBP | fructose-1,6-bisphosphatase II sedoheptulose-1,7-bisphosphatase [EC 3.1.3.11 3.1.3.37] |
| **K15521** | *mshA* | D-inositol-3-phosphate glycosyltransferase [EC 2.4.1.250] |
| **K19003** | *mgdA* | 1,2-diacylglycerol 3-beta-glucosyltransferase [EC 2.4.1.336] |

**Table S2. Genes with conserved synteny in picocyanobacteria that were used to construct the species tree in Figure 1**. The first column provides the KEGG identifier (KO), the second column lists the gene name (or protein name when KEGG does not provide a gene name for the KO), and the third column provides a description of the gene function.

**Supplemental Figure Legends**

**Figure S1.** MAFFT (V7.54; (1)) alignment (top) of internal transcribed spacer (ITS) regions derived from *Prochlorococcus* isolates representing established clades (LLI, LLII/III, LLIV) and novel grades (LLVII, LLVIII) created using default settings in the Geneious software package (V10.2.6;(2)). A percent identity matrix for the alignment is also included (bottom).

**Figure S2.** Photophysiology comparison with NATL2A (LLI clade).  Light-dependent growth rates (A), *in vivo* absorption spectra for log phase cultures of different strains growing at 5.7 - 5.8 μmol photons m^-2^ s^-1^ (B), and flow cytometry parameters red fluorescence (C), yellow fluorescence (D), forward scatter (E) for NATL2A - a well studied LL-adapted strain of *Prochlorococcus* (3–5) - for comparison with the novel strains reported in this study. Open circles (E) denote the light level at which the maximum growth rate was achieved for each strain. Data for MIT1314, MIT1223, and MIT1327 are reproduced from figures 2, 4, and 5.

**Figure S3.** HPLC-Orbitrap-MS chromatograms are shown as extracted ion chromatograms (EICs) of identified pigments in *Prochlorococcus* MIT1314 (HLII clade) acclimated to an irradiance of 21.6 µmol photons m^-2^ s^-1^.

**Figure S4.** Molecular structures of pigments identified in *Prochlorococcus* strains MIT1327, MIT1300xe, MIT1223, and MIT1314.

**Figure S5.** HPLC-Orbitrap-MS chromatogram shown as extracted ion chromatogram (EIC) of an unknown pigment ([M+H]^+^ at *m/z* 553.44039) in *Prochlorococcus* MIT1314 acclimated to an irradiance of 21.6 µmol photons m^-2^ s^-1^ (A). The proposed sum formula (C_40_H_56_O) based on the accurate mass in full scan is also shown. MS^2^ spectrum of the unknown carotenoid ([M+H]^+^ at *m/z* 553.44039) (B). We observed a dominant fragment ion at *m/z* 123.117 in the MS^2^ spectrum, which is typically associated with *α*-carotene (6) as the fragment represents the ε ring. The second dominant fragment at *m/z* 105 is usually associated with epoxy xanthophylls (6).

**Figure S6.** HPLC-Orbitrap-MS chromatogram shown as extracted ion chromatogram (EIC) of an all-*trans*-retinal standard ([M+H]^+^ at *m/z* 285.22129) and an EIC of the same mass in *Prochlorococcus* MIT1314 acclimated to an irradiance of 21.6 µmol photons m^-2^ s^-1^. The match of the accurate mass together with the retention time indicates the presence of all-*trans*-retinal in *Prochlorococcus*, but further identification is needed to confirm this identification. Due to its low abundance, we were not able to obtain MS^2^ spectra of the putative retinal.

**Figure S7:** Trees of picocyanobacterial genes that contain the pfam PF00421 (Photosystem II protein) domain. (A) Sequences were extracted based on the presence of the PF00421 domain. Colors represent assigned gene annotations as used in panels B to G except for IsiA, which is shown in Supplementary Figure 8. PcbE/F proteins containing weak hits to PF00421 were excluded from the tree. For PsbB/C, 100 sequences were chosen at random, as they are all monophyletic. (B-G) trees of individual genes with tip colors depicting ecotype as shown in the legend. (B) PcbA mostly fits the species phylogeny and shows very different alleles for Basal (LL-IV, and AMZ-I), LL, and HL clades. (C) PcbB is found in LL only. MIT1223 together with LL-I. LL-I also had a duplication, which MIT1223 lacks. (D) MIT1223 embedded in LL-VII. (E) agrees with the species tree. (F) MIT1223 closest to LL-I. (G) MIT1223 has a divergent allele, dissimilar to both LL-VII and LL-I.

**Figure S8:** Trees of phycobilisome associated genes. Tip colors represent ecotype as denoted in the legend. (A) *Prochlorococcus* allele is completely different from *Synechococcus*, except for AMZ-III, and II, which share the *Synechococcus* allele. (B) MIT1223 embedded in LL-VII. *Prochlorococcus* allele is completely different from *Synechococcus*, except for AMZ-III, and II, which share the *Synechococcus* allele. (C) MIT-1223 has a divergent allele, dissimilar to both LL-VII and LL-I. (D) Duplication in *Synechococcus*. AMZ-II and III acquired both copies. LL-VII and LL-II,III have a different allele that diverged from the ancestor of *Synechococcus* genes before their duplication. (E) HL allele is completely different from rest. Basal *Prochlorococcus* (AMZ-I, LL-IV, LL-II.III, LL-I, and LL-VII) have a different allele than *Synechococcus*, which has a duplication that is shared with AMZ-II and III. (F) MIT1223 has a divergent allele, dissimilar to both LL-VII and LL-I. Basal *Prochlorococcus* (AMZ-I to AMZ-III, and LL-IV) are embedded in *Synechococcus* diversity. (G) *Prochlorococcus* portion agrees with the species tree. AMZ-II and AMZ-III each have a different *Synechococcus* allele. (H) Duplication in *Synechococcus*. AMZ-II, and II have both *Synechococcus* alleles. *Prochlorococcus* diversity agrees with the species tree, and branches off from one of the *Synechococcus* alleles. (I) Three clades in *Synechococcus*. AMZ-II, and AMZ-III each have a different *Synechococcus* allele. MIT1223 embedded in LL-VII diversity. (J) *Prochlorococcus* completely separated from *Synechococcus*. Complex history in *Pro*. MIT1223 and some LL-VII share the LL-I allele.

**Figure S9.** Histograms showing percent deviation from target light level for irradiance measurements taken over the course of light-dependent growth experiments.

**Supplemental References**

1. Katoh K, Standley DM. 2013. MAFFT Multiple sequence alignment software version 7: improvements in performance and usability. Molecular Biology and Evolution 30:772–780.

2. Kearse M, Moir R, Wilson A, Stones-Havas S, Cheung M, Sturrock S, Buxton S, Cooper A, Markowitz S, Duran C, Thierer T, Ashton B, Meintjes P, Drummond A. 2012. Geneious Basic: An integrated and extendable desktop software platform for the organization and analysis of sequence data. Bioinformatics 28:1647–1649.

3. Scanlan DJ, Hess WR, Partensky F, Newman J, Vaulot D. 1996. High degree of genetic variation in *Prochlorococcus* (Prochlorophyta) revealed by RFLP analysis. European Journal of Phycology 31:1–9.

4. Biller SJ, Berube PM, Lindell D, Chisholm SW. 2015. *Prochlorococcus*: the structure and function of collective diversity. Nat Rev Micro 1–15.

5. Berube PM, Rasmussen A, Braakman R, Stepanauskas R, Chisholm SW. 2019. Emergence of trait variability through the lens of nitrogen assimilation in *Prochlorococcus*. Elife 8:10679.

6. Rivera SM, Christou P, Canela-Garayoa R. 2014. Identification of carotenoids using mass spectrometry. Mass Spec Rev 33:353–372.
